# Consumption of Lean Fish Reduces the Risk of Type 2 Diabetes Mellitus: A Prospective Population Based Cohort Study of Norwegian Women

**DOI:** 10.1371/journal.pone.0089845

**Published:** 2014-02-24

**Authors:** Charlotta Rylander, Torkjel M. Sandanger, Dagrun Engeset, Eiliv Lund

**Affiliations:** 1 Department of Community Medicine, UiT The Arctic University of Norway, Tromsø, Norway; 2 NILU, Fram- High North Research Centre for Climate and the Environment, Tromsø, Norway; College of Pharmacy, University of Florida, United States of America

## Abstract

**Background:**

The effects of fish consumption and n-3 fatty acids on type 2 diabetes mellitus (T2DM) have recently been debated.

**Objective:**

We explored the risk of T2DM in relation to consumption of lean fish, fatty fish, fish products and total fish as well as cod liver oil supplements in a representative sample of Norwegian women.

**Design:**

This was a prospective population based cohort study in 33740 women free of T2DM, stroke, angina or heart attack and with detailed information on important co-variates and dietary intake at baseline. Risk ratios and corresponding 95% CI were estimated using Poisson regression with log-person time as offset.

**Results:**

Lean fish consumption was inversely associated with T2DM compared to zero intake. Risk ratios and 95% CI for intake of 75 and 100 g lean fish per day were 0.71 (0.51, 0.98) and 0.67 (0.46, 0.98), respectively. There was no effect of intake of fatty fish, fish products, total fish or use of cod liver oil supplements on the risk of T2DM.

**Conclusion:**

Lean fish consumption of 75–100 g/d had a beneficial effect on T2DM. It remains unclear whether lean fish in itself has a protective effect on T2DM or that lean fish consumers have a protective life-style that we were not able to take into account in this study. Unfavorable effects of fatty fish consumption or use of cod liver oil supplements on T2DM were not observed.

## Introduction

Type 2 diabetes mellitus (T2DM) is increasing in epidemic proportions worldwide and is becoming a global health challenge. In 1980, approximately 153 million people had diabetes and by 2008, that number was more than doubled [1]. It has been estimated that the global burden of diabetes will further increase to more than 550 million by 2030 [2], thus, prevention of the disease is of great public health interest.

Even though the causes of T2DM are not fully understood, a number of risk factors are well known and include among others: overweight and obesity, family history of diabetes, age, impaired glucose tolerance, sedentary lifestyle, hypertension and history of gestational diabetes. As T2DM is closely linked to overweight and obesity, there has been an increasing focus on the effect of different diets on the prevention and management of T2DM [3]. T2DM and cardiovascular diseases (CVD) have many of the same risk factors in common. Since consumption of fatty fish that contain long chain n-3 fatty acids has showed beneficial effects on the risk of CVD as well as on blood lipids profiles [4], one could expect fish consumption reducing the risk of T2DM as well. Recently, a number of prospective studies have explored associations between fish consumption and risk of T2DM, with conflicting findings [5]–[8]. One limitation of some of these studies has been the lack of distinction between fatty and lean fish. Several systematic reviews and meta-studies have concluded that there are indications of geographical differences in observed effects of fish consumption, with studies from Asia showing a protective effect whereas studies from North America/Europe indicating an increased risk of T2DM with fish consumption [9], [10]. In contrary, a recent nested case-cohort study across eight European countries concluded no effect of total fish consumption on T2DM and indicated a protective effect of fatty fish consumption on the development of T2DM [11].

Fish, and particularly lean fish, is a major part of the Norwegian diet, especially for people living along the Norwegian coastline. Age-adjusted mean intake of total fish was 63 g/day for women residing along in the north or the west coast of Norway [12]. For women living in southeast Norway the corresponding number were 42 g/d. Even though there are geographical differences, Norwegian middle-aged women consume almost twice as much fish as women in the other Scandinavian countries and almost five times as much as women from the Netherlands [12]. The Norwegian Directorate of Health recommends people in general, and young people particular, to consume at least two servings of fish per week. A large proportion (45%) of Norwegian middle-aged women also uses cod liver oil as dietary supplements regularly [13]. Cod liver oil supplements, as well as fatty fish, contain long-chain n-3 fatty acids. A number of clinical trials have addressed the relationship between long chained n-3 fatty acids and the glucose metabolism. Some have indicated reduced insulin sensitivity among obese or diabetic subjects after treatment with n-3 supplements [14], [15], however a recent meta-analysis of 11 clinical trials concluded no effect [16].

As Norwegian women have a high intake of fish as well as a regular use of n-3 supplements, this study was conducted to explore the risk of T2DM in relation consumption of lean fish, fatty fish, processed fish products, total fish and cod liver oil supplements.

## Subjects and Methods

### The Norwegian Woman and Cancer Study

The Norwegian Women and Cancer Study (NOWAC) is a national representative cohort study that was initiated in 1991 with the aim of exploring associations between lifestyle and cancer among Norwegian women. Detailed information about study design, population and procedures has been described elsewhere [17]. The external validity of NOWAC has also been thoroughly validated [18]. Currently, more than 170 000 Norwegian women aged 30–70 years have been enrolled in the study. All participants have answered one to four questionnaires about their current health status and different lifestyle habits, including diet. The questionnaires have been distributed in series and the questions in the different series have varied depending on age group and hypothesis tested; however, some questions have been common in all series. Between 2003 and 2007, approximately 50 000 of the NOWAC participants also donated a blood sample. The latter constitute the NOWAC postgenome cohort [19].

### Ethics Statement

The NOWAC study was approved by the Regional Committee for Medical Research Ethics and the Norwegian Data Inspectorate. All participants gave written informed consent.

### NOWAC Food Frequency Questionnaire

The majority of the NOWAC questionnaires contain detailed questions on dietary habits. The participants were asked to record how often they consumed more than 90 different foodstuffs during the preceding year. Information about portion size was also included. The food frequency questionnaire (FFQ) has special emphasis on fish consumption. The NOWAC FFQ has been thoroughly validated by 24 h recalls [20], a test-retest study [21] and against serum phospholipid fatty acid composition as biomarkers of fish consumption [22]. The validation studies showed that the NOWAC FFQ has good ability to rank subjects according to food eaten frequently. The reproducibility of the NOWAC FFQ was in the same range as similar instruments. The validation study on fish consumption underlined the importance of distinction between fatty and lean fish and the inclusion of portion sizes in order to achieve high quality data.

### Cross-sectional Validation of Self-reported Diabetes

In the NOWAC questionnaire, the participants are asked if they have diabetes (yes/no) and at what age they got the diagnosis. There is no distinction between different types of diabetes. In order to ensure proper endpoints, we conducted a cross-sectional validation study of self-reported diabetes based on information from 47603 subjects. Between 2002 and 2007, these subjects answered their second or third questionnaire. Some of them also donated a blood sample. Criteria for being included in the validation study were a positive answer on self-reported diabetes. In total 1066 (2.2%) women reported having diabetes, of which a random sample of 400 subjects was drawn. Of the randomly selected 400 subjects, 226 (56.5%) had donated a blood sample. The 400 study subjects were identified by Statistics Norway and we were provided with full name and home address to the participants. Twenty-one women were either diseased or emigrated; therefore, the total number included in the validation study was 379.

The 379 subjects were invited to participate in the study through an invitational letter sent to their home address. The participants were asked to confirm their diabetes diagnosis. They were also asked what year they got their diagnosis and how they were treated for the disease. Additionally, we asked for permission to contact their general physician (GP) in order to confirm what type of diabetes the participants had (type 1, type 2 or other).

The GPs were contacted through a letter to their office where we asked for confirmation of the diabetes diagnosis, as well as year of diagnosis and type of diabetes. The validation study was approved by the Regional Committee for Medical Research Ethics.

Among the 379 subjects included in the validation study, the total response rate was 58%, corresponding to 217 subjects. Blood donors were more likely to respond (65%) than the non-donors (47%). Among the 211 subjects willing to participate in the study, 177 (84%) confirmed having T2DM. The remaining 34 subjects (16%) reported either T1DM (7.5%), gestational diabetes (1.4%), Mody 3 (0.5%) or other diagnosis (0.5%). Thirteen subjects (6.1%) claimed never having any form of DM. Thus, the positive predictive value (PPV) of the questionnaire in regards to T2DM was 84%. PPV for all forms of diabetes was 93%. Excluding those refuting having diabetes showed that 89.4% of all diabetes cases were T2DM. Self-reported mean age at time for diagnosis for confirmed T2DM cases were 48 years (min-max: 36–61 years). For those subjects claiming having T1DM, the self-reported mean age at time for diagnosis were 44 years (min-max: 35–57 years). Only subjects confirming their T2DM diagnosis allowed us to contact their GP. The response rate among GPs was 82%. The GPs confirmed T2DM in 141 of 146 cases and disproved the diagnosis for five subjects. Thus, PPV of the second confirmation from the subjects was 97%.

Of the 177 women confirming their diabetes diagnosis, 39 had missing information on self-reported diabetes at their previous questionnaire. Of these 39, we received information about the year of diagnosis from 30 GPs. When comparing year of diagnosis to date of baseline questionnaire, we found that 25 (83.3%) were incident cases, i.e., missing information on self-reported diabetes was equal to “No”. For 1 (3.3%) subject, year of diagnosis were prior to date of first questionnaire and for the remaining four (13.3%) subjects, it was unclear as year of diagnosis and date of first questionnaire were less than six months apart.

### The Prospective Analysis of Fish Consumption and Risk of Type 2 Diabetes

#### Study population

A total of 48232 women were included in the prospective analysis of fish consumption and risk of T2DM. The study subjects answered the baseline questionnaire between 1996 and 1998. Between 2002 and 2005, the subjects were followed-up with a repeated questionnaire, thus follow-up time were defined by date of questionnaire at baseline and date of questionnaire at follow-up. As we had no valid confirmation of date of diagnosis for the cases, their follow-up time was defined as half the time between the two questionnaires.

At baseline, there were 542 self-reported prevalent cases of diabetes, which were excluded from further analyses. In addition, 2132 women were excluded as they were not asked dietary questions at baseline and 86 subjects that were not asked questions on disease history. We made further exclusions for baseline missing information on key co-variates including body mass index (BMI, n = 738), physical activity (n = 2739) and smoking (n = 1620). We further restricted the analyses to only those with a certain detailed dietary questionnaire at baseline (n = 34845). Subjects being in the top 1% or bottom 1% of the ratio of total daily energy intake and basal metabolic rate (calculated from weight, height and age at baseline) were excluded (n = 698). We further excluded subjects with history of stroke, angina or heart attack (n = 407). There were 4811 subjects with missing information on hypertension (yes/no). Including their missing answers as negative answers did not change the effect estimates substantially, and thus they were included in the analysis. In total, 33740 subjects were included in the prospective analyses.

#### Exposure variables

Daily intake (g/day) of fatty fish, lean fish, processed fish products, total fish and use of cod liver oil supplements were explored. Fatty fish included salmon, trout, herring, mackerel, wolffish, redfish and flounder whereas lean fish comprised cod, saithe, pollock and haddock. Fish products included fish fingers (coated in bread crumbles), minced fish products (fish cakes, fish balls etc.) and fish au gratin. These are mainly made from lean fish and contain on average 30–50% fish. Even though fish products contain lean fish, this variable was evaluated separately since it consists of a large proportion of flour, milk and fat as well. Total fish included fatty fish, lean fish, fish products, preserved fish for bread, fish liver, fish roe and crustaceans. Use of cod liver oil supplements was assembled into two categories (yes/no).

#### End-point

Of the 33740 subjects included in the prospective analyses, there were 479 reported incident cases of diabetes at follow-up. However, only 335 of these subjects had a negative answer on diabetes at baseline and a positive at follow-up. The remaining 144 subjects had missing answers on the diabetes question at baseline and a positive answer at follow-up. For the prospective analyses, missing information on diabetes were interpreted as a negative answer, thus, 479 cases were included in the study. The cases are hereafter referred to as T2DM cases.

### Statistical Methods

The freely available statistical software R (version 3.0.0) with the packages Epi, splines and survival was used for the statistical analyses (http://www.cran.r-project.org). Differences in baseline characteristics between diabetes cases and the non-diabetic subjects were evaluated using analyses of variance and chi square test. Risk ratios and their corresponding 95% CI were estimated using Poisson regression with the log-person time as offset and the use of natural splines with 4 degrees of freedom for the exposure variables of interest. When Poisson regression is used for modeling rates, the logarithm of the time unit (here person-years) is added to the model as a fixed variable to take into account the various person-time of the study subject. This is called an “offset”. Natural splines split the exposure variables into segments and fit a cubic function for each segment. Within each segment, the function is estimated to be constant. By extracting the knots (the cut-off values between segments) and the boundary knots (the extreme values of the exposure variable) for the splines, a contrast matrix was constructed, which was multiplied with the effects estimates of the splines. This way it is possible to predict the effect of the exposure variables on T2DM at specified points, here every 25 g intake per day. The effect were predicted well within the range of the original data set as the effect estimates becomes more uncertain close to extremes, due to the right-skewed distribution of the fish variables. Due to the lower consumption of fatty fish in the study group, risk ratios were only predicted up to 50 g/d compared to 75 g/d or 225 g/d for the other exposure variables.

We evaluated the effect of all exposure variables and co-variates separately, age-adjusted and in multivariate models. The following co-variates were explored : body mass index (BMI, assembled into three categories: <25 kg/m^2^, 25–30 kg/m^2^, >30 kg/m^2^), physical activity (recorded in the questionnaire on a scale ranging from 1 to 10 and assembled into three categories; “low” (1–3), “moderate” (4–7) and “high” (8–10), years of education (assembled into three categories “<10 years”, “10–12 years”, “>12 years”), total energy intake (kcal/d), alcohol intake (assembled into four categories; “0 g/d”, “1–50 g/d”, “51–100 g/d”, “>100 g/d”), smoking status (assembled into three categories; “never”, “former”, “current”), self-reported hypertension (yes/no), calculated daily intake of saturated fat (g/d) and carbohydrates (g/d). The models were also evaluated for adjustment of the various types of fish consumed. Analysis of variance was used for assessing whether inclusion of an extra co-variate improved the model or not. The residual deviance was computed for the full and the reduced model and compared. The model with lowest residual deviance was considered having the best model fit. If there was no significant evidence for a better model fit when including an additional co-variate, the simplest model was preferred.

## Results

### Fish Consumption and Risk of Type 2 Diabetes

Baseline characteristics of the total study population, the incident T2DM cases and the non-diabetic subjects are provided in Table 1. Mean age at enrollment was 47.9 years (min-max: 40–55), 31.9% were current smokers and 55.2% had more than 12 years of school. The majority (65.0%) of women had BMI<25 kg/m^2^. Diabetes cases were significantly different from the non-diabetics in a number of ways: almost 80% of diabetes cases were characterized as overweight (>25 kg/m^2^) or obese (>30 kg/m^2^). In addition, a larger proportion of diabetes cases reported low physical activity (22.5% vs. 11.9%), higher prevalence of hypertension (28.6% vs. 9.3%) and more former or current smokers than the non-diabetics. The prospective diabetes cases were further characterized by less education, lower alcohol consumption and slightly lower intake of total carbohydrates than the non-diabetics at baseline.

**Table 1:** Self-reported baseline characteristics of the total study population (n = 33740), the incident diabetes cases (n = 479) and the non-diabetic subjects (n = 33261).

		Total (n = 33740)	Diabetes cases (n = 479)	Non-diabetic subjects (n = 33261)	p-value^a^
**Age (years)**	Mean age at enrollment	47.9	48.7	47.9	2.3e-6
	Min-max	40–55	40–55	40–55	
**Energy intake (kcal/d)**	Mean	1683	1668	1683	3.9e-1
	Median	1657	1619	1657	
	Min-Max	676–3632	774–3082	676–3632	
**Carbohydrates (g/d)**	Mean	197	192	197	2.0e-2
	Median	194	186	194	
	Min-Max	37.8–420	74.4–381	37.8–420	
**Saturated fat (g/d)**	Mean	25.8	25.5	25.8	2.9e-1
	Median	24.7	24.0	24.7	
	Min-Max	1.05–90.3	4.8–57.2	1.05–90.3	
**BMI (%)**	<25	65.0	21.9	65.7	2.2e-16
	25–30	27.3	36.4	27.2	
	>30	7.6	34.9	7.2	
**Years of education (%)** b	<10	20.4	27.2	20.3	1.5e-5
	10–12	24.4	28.0	24.3	
	>12	55.2	44.8	55.4	
**Physical activity (%)**	Low	12.0	22.5	11.9	8.0e-12
	Moderate	75.1	67.2	75.2	
	High	12.9	10.2	12.9	
**Smoking status (%)**	Never	37.7	32.1	37.8	4.0e-2
	Former	31.4	33.8	31.4	
	Current	31.9	34.0	30.8	
**Hypertension (%)**	No	90.5	71.4	90.7	2.2e-16
	Yes	9.5	28.6	9.3	
**Alcohol consumption (%)**	0 g/d	18.6	27.6	18.4	4.4e-7
	1–50 g/d	40.0	40.5	40.0	
	50–100 g/d	29.1	23.4	29.2	
	>100 g/d	12.3	10.8	12.4	

a The p-value refers to the difference between diabetic and non-diabetic individuals.

b Missing information on education, n = 1148.

Mean intake of total fish in the study group was 93 g/d (median 83 g/d, min-max: 0–567 g/d), dominated by intake of fish products (mean: 37 g/d, median: 32 g/d, min-max: 0–255 g/d) and lean fish (mean 29 g/d, median: 24 g/d, min-max: 0–244 g/d). Mean intake of fatty fish was 15 g/d (median: 10 g/d, min-max: 0–260 g/d). Forty-five percent of the study group used cod liver oil supplements at least once a week.

Crude and age-adjusted risk ratios (RR) of T2DM in relation to the exposure variables and the co-variates are listed in Table 2. Body mass index, hypertension, physical activity, alcohol consumption and smoking were strongest associated with T2DM (Table 2).

**Table 2:** Crude risk ratios (RR) with 95% confidence intervals (95% CI) and age-adjusted RR (95% CI) of T2DM in relation to a number of co-variates and exposure variables.

	RR _crude_ (95% CI)	p-value	RR_age-adjusted_ (95% CI)	p-value
**Age (years)**	1.04 (1.02, 1.06)	1.2e-4	–	–
**BMI (kg/m^2^)**				
<25 (n = 21946)	1.00		1.00	
25–30 (n = 9222)	4.38 (3.45, 5.55)	<2e-16	4.31 (3.40, 5.47)	<2.0e-16
>30 (n = 2572)	15.4 (12.1, 19.6)	<2e-16	15.2 (11.9, 19.3)	<2.0e-16
**Physical activity**				
Low (n = 4065)	1.00		1.00	
Medium (n = 25326)	0.47 (0.38, 0.59)	1.5e-11	0.48 (0.39, 0.60)	4.7e-11
High (n = 4349)	0.42 (0.30, 0.59)	4.3e-7	0.43 (0.31, 0.60)	9.2e-7
**Hypertension**				
No (n = 30520)	1.00		1.00	
Yes (n = 3220)	3.89 (3.19, 4.74)	<2e-16	3.74 (3.06, 4.57)	<2.0e-16
**Smoking status**				
Never (n = 12720)	1.00		1.00	
Former (n = 10603)	1.27 (1.02, 1.59)	3.0e-2	1.30 (1.04, 1.62)	2.0e-2
Current (n = 10417)	1.31 (1.05, 1.63)	2.0e-2	1.35 (1.08, 1.68)	8.0e-3
**Education (years)**				
<10 (n = 6652)	1.00		1.00	
10–12 (n = 7948)	0.85 (0.67, 1.09)	2.0e-1	0.88 (0.69, 1.13)	3.2e-1
>12 (n = 17992)	0.60 (0.48, 0.75)	5.6e-6	0.63 (0.51, 0.79)	6.5e-5
**Alcohol consumption (g/d)**				
0 (n = 6257)	1.00		1.00	
<50 (n = 13507)	0.68 (0.54, 0.85)	6.0e-4	0.69 (0.55, 0.86)	1.1e-3
51–100 (n = 9817)	0.53 (0.41, 0.69)	9.4e-7	0.54 (0.42, 0.70)	2.2e-6
>100 (n = 4159)	0.46 (0.32, 0.65)	1.2e-5	0.47 (0.33, 0.67)	2.4e-5
**Cod liver oil supplements**				
No (n = 18072)	1.00		1.00	
Yes (n = 14763)	0.77 (0.64, 0.93)	6.8e-3	1.04 (1.02, 1.07)	3.4e-3
**Carbohydrates (per 50 g/d)**	0.89 (0.82, 0.98)	1.2e-2	0.90 (0.82, 0.98)	1.8e-2
**Saturated fat(per 10 g/d)**	0.96 (0.86, 1.06)	3.9e-1	0.97 (0.87, 1.07)	5.3e-1
**Total energy intake (per 500 kcal)**	0.95 (0.85, 1.06)	3.4e-1	0.96 (0.86, 1.07)	4.5e-1
**Lean fish (per 25 g/d)**	1.02 (0.95, 1.11)	5.7e-1	1.01 (0.93, 1.10)	7.9e-1
**Fatty fish (per 25 g/d)**	1.13 (1.01, 1.28)	4.0e-2	1.11 (0.99, 1.25)	7.8e-2
**Fish products (per 25 g/d)**	1.04 (0.95, 1.13)	4.1e-1	1.04 (0.96, 1.13)	3.6e-1
**Total fish (per 25 g/d)**	1.04 (1.00, 1.09)	2.8e-2	1.04 (1.00, 1.08)	5.1e-2

**Figure 1:**
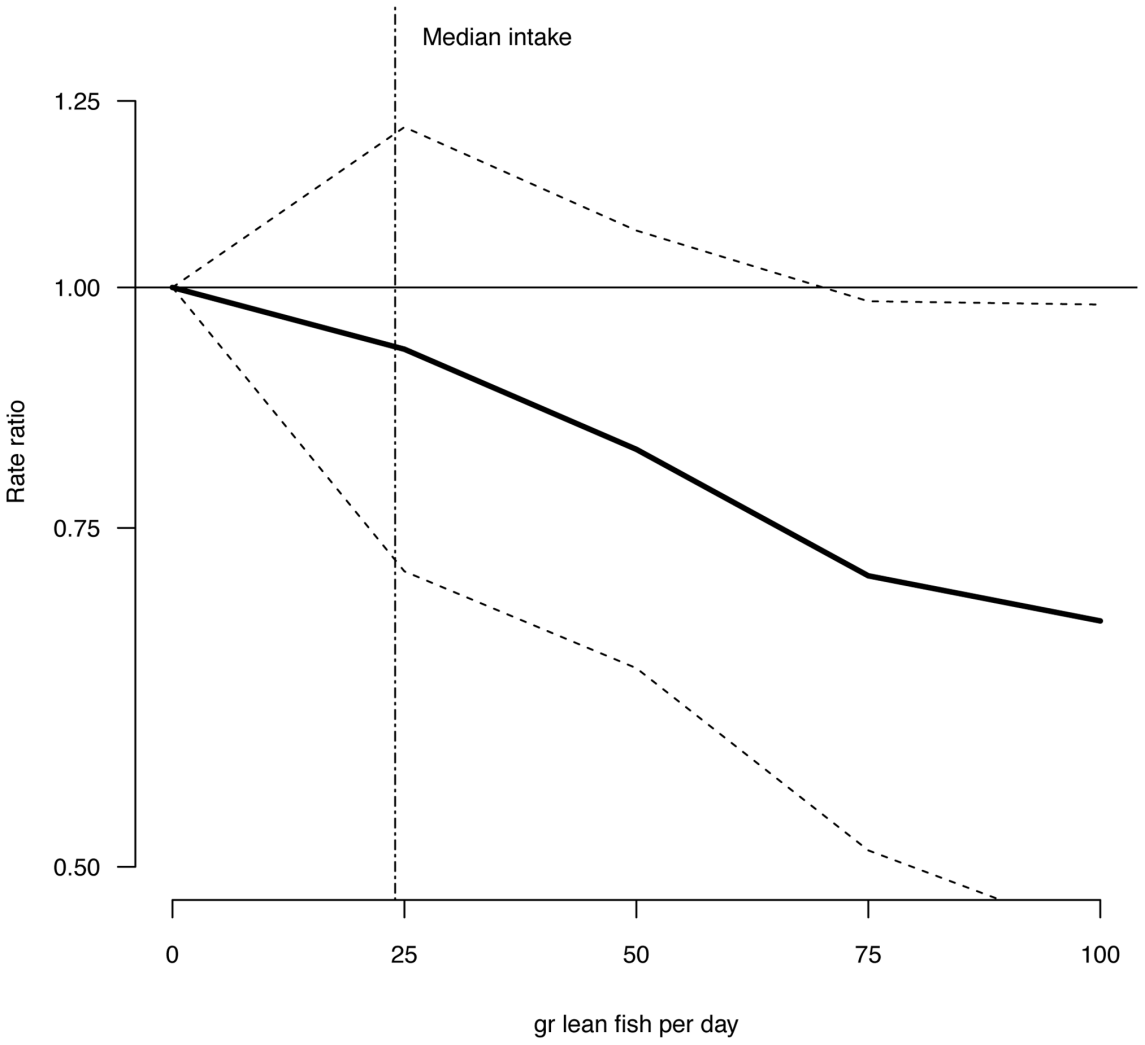
Risk ratios (RR) and 95% CI of T2DM by lean fish consumption. RRs are indicated by the solid line and the 95% CI by the dashed horizontal lines. Numbers are adjusted for BMI, hypertension, smoking status, age and physical activity. Median intake of lean fish in the study group is indicated by the dashed vertical line.

**Figure 2:**
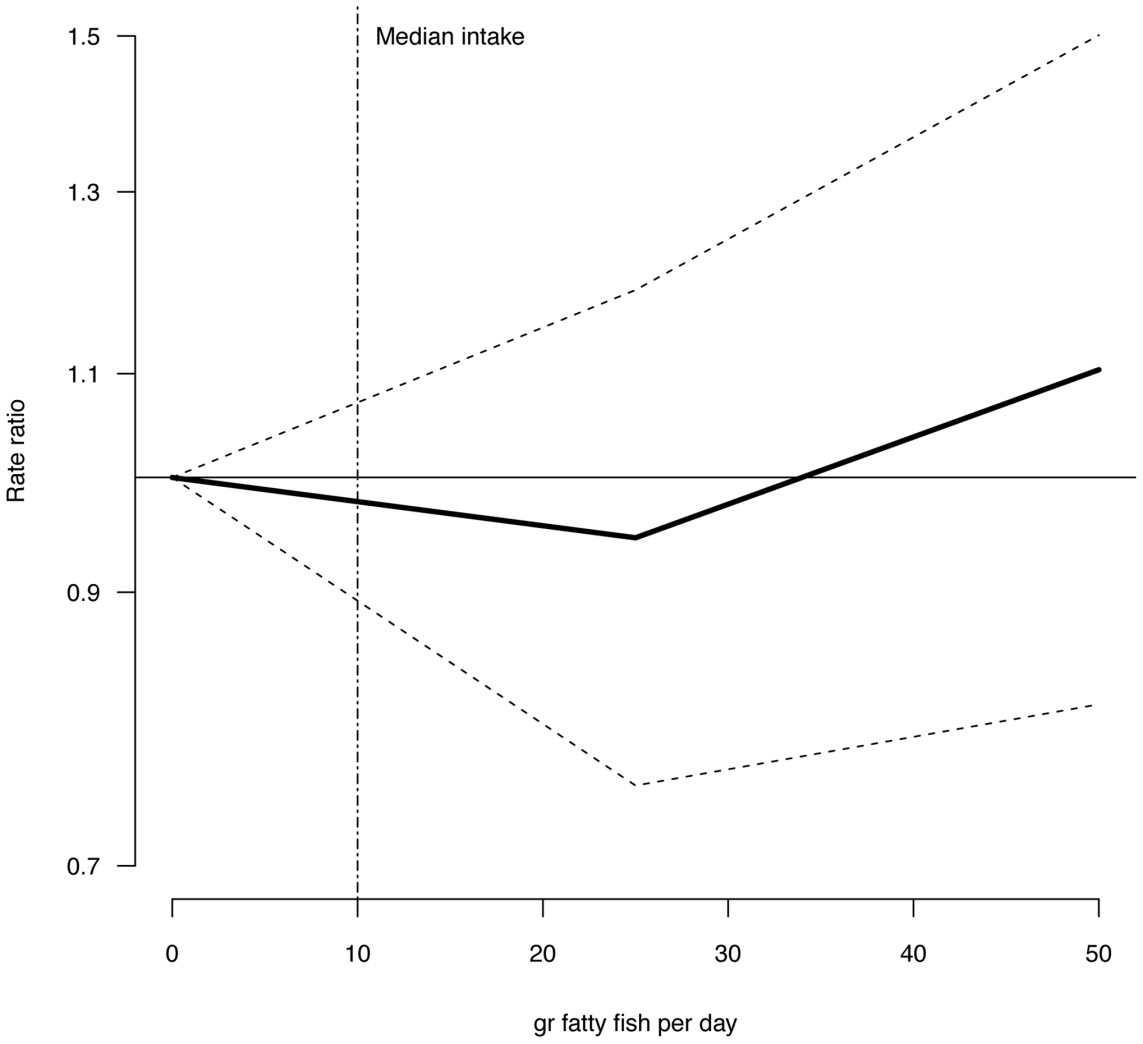
Risk ratios (RR) and 95% CI by fatty fish consumption. RRs are indicated by the solid line and the 95% CI by the dashed horizontal lines. Numbers are adjusted for BMI, hypertension, smoking status, age and physical activity. Median intake of fatty fish in the study group is indicated by the dashed vertical line.

**Figure 3:**
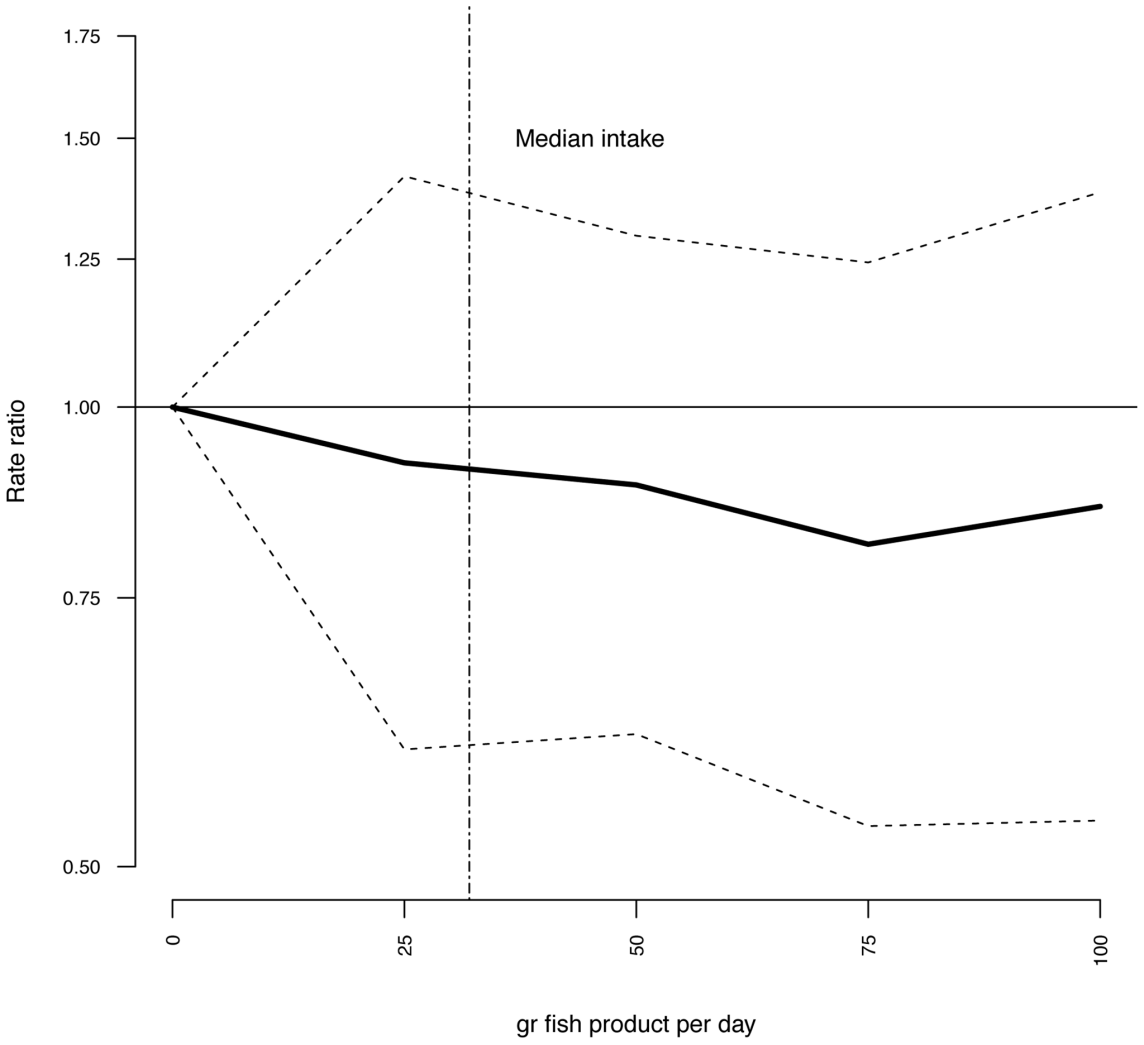
Risk ratios (RR) and 95% CI by fish product consumption. RRs are indicated by the solid line and the 95% CI by the dashed horizontal lines. Numbers are adjusted for BMI, hypertension, smoking status, age and physical activity. Median intake of fish products in the study group is indicated by the dashed vertical line.

**Figure 4:**
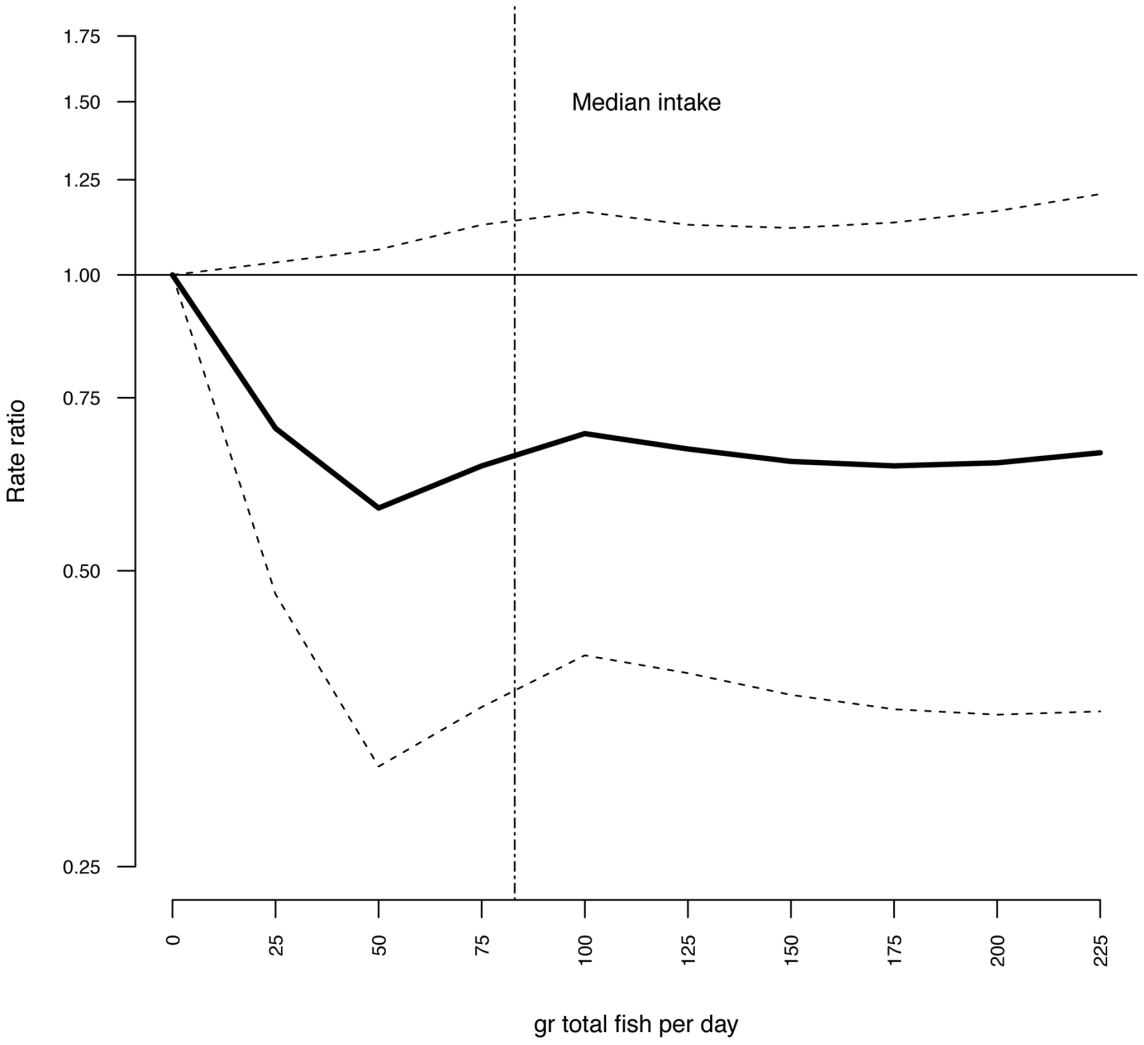
Risk ratios (RR) and 95% CI by total fish consumption. RRs are indicated by the solid line and the 95% CI by the dashed horizontal lines. Numbers are adjusted for BMI, hypertension, smoking status, age and physical activity. Median intake of total fish in the study group is indicated by the dashed vertical line.

Multivariate adjusted RR for intake of lean fish, fatty fish, fish products, total fish, and cod liver oil supplements in relation to risk of T2DM are illustrated in Figure 1–4 and listed in Table 3. Consuming 75–100 g of lean fish per day, reduced the risk of T2DM by approximately 30% (Table 3 and Figure 2). Intake of fatty fish, fish products, total fish or cod liver oil supplement had no effect on T2DM (Table 3, Figure 1–4).

**Table 3:** Risk of T2DM in relation to fish consumption in the study population (n = 33740).

Lean fish (g/d)	RR (95% CI)^a^	p-value
0	1.00	
25	0.93 (0.71, 1.21)	0.59
50	0.82 (0.63, 1.07)	0.15
75	0.71 (0.51, 0.98)	0.04
100	0.67 (0.46, 0.98)	0.04
**Fatty fish (g/d)**		
0	1.00	
25	0.95 (0.75, 1.19)	0.63
50	1.1 (0.81, 1.50)	0.53
**Fish products (g/d)**		
0	1.00	
25	0.92 (0.60, 1.42)	0.70
50	0.89 (0.61, 1.29)	0.54
75	0.81 (0.53, 1.24)	0.34
100	0.86 (0.54, 1.38)	0.54
**Total fish (g/d)**		
0	1.00	
25	0.70 (0.47, 1.03)	0.07
50	0.58 (0.32, 1.06)	0.08
75	0.64 (0.36, 1.12)	0.12
100	0.69 (0.41, 1.16)	0.16
125	0.67 (0.39, 1.12)	0.13
150	0.65 (0.37, 1.12)	0.12
175	0.64 (0.36, 1.13)	0.12
200	0.64 (0.36, 1.16)	0.14
225	0.66 (0.36, 1.21)	0.18
**Use of cod liver oil supplements**		
No	1.00	
Yes	0.95 (0.79, 1.14)	0.56

Risk ratios (RR) and 95% confidence intervals (95% CI) are provided.

a Adjusted for age, BMI, smoking status, physical activity and hypertension.

### Prevalence and Incidence of Diabetes in NOWAC

**Figure 5:**
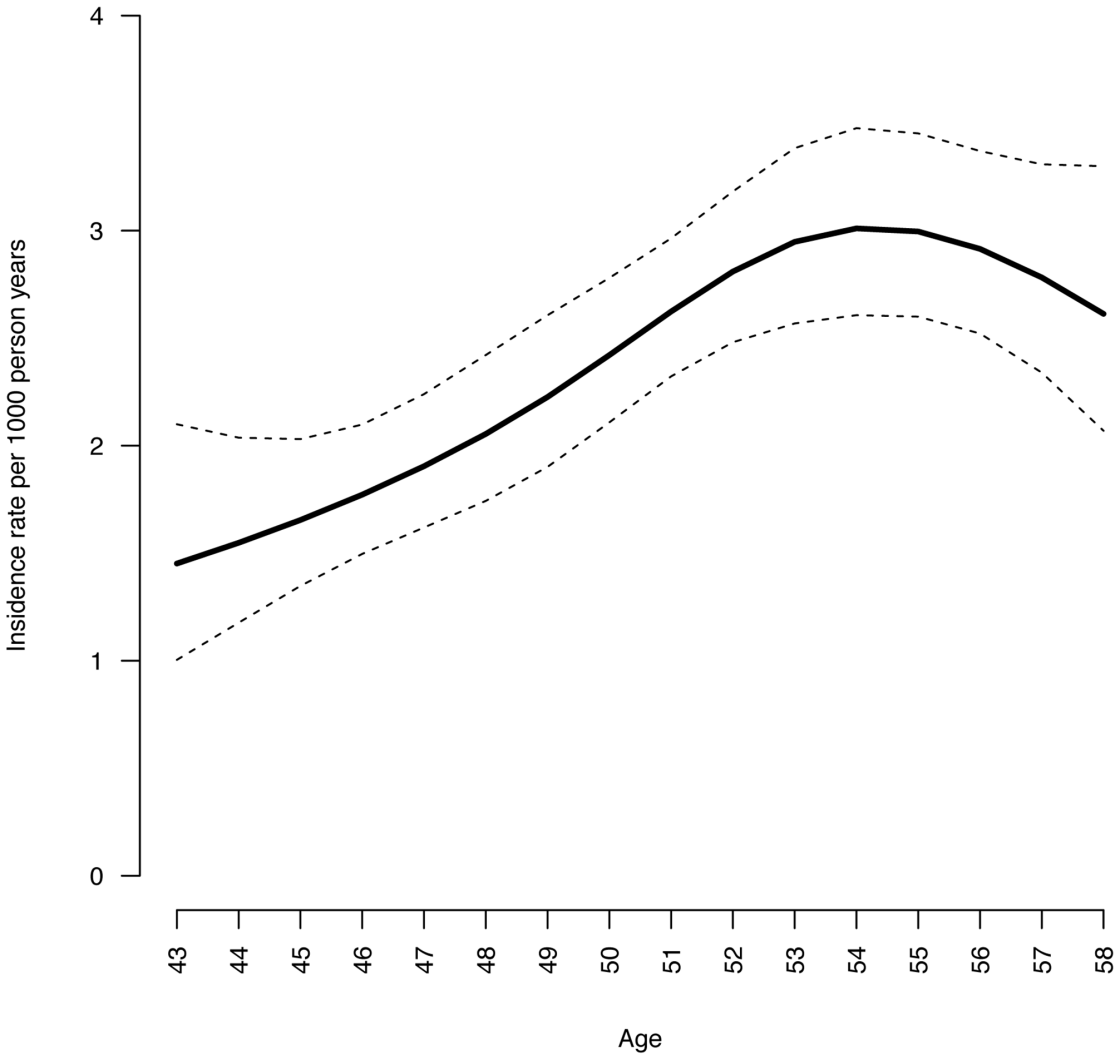
Incidence rate of DM by age in the NOWAC cohort.

Prior to any exclusions made, the study population consisted of 48232 subjects of whom 1232 reported having DM at follow-up (year 2002–2007). Thus, the prevalence of all forms of DM at follow-up was 2.6%. After the exclusions, 479 incident T2DM cases were identified in the prospective study group of 33740 subjects and 199054.4 person years. Thus, the overall incidence rate per 1000 person years was 2.41 (95% CI: 2.20, 2.63). Figure 5 shows the incident rate of T2DM in the study population by age.

We conducted a sensitivity analyses on the incidence proportion by excluding all subjects with missing answers on self-reported diabetes. This way, we identified 335 incident diabetes cases in a total study group of 24429 subjects, contributing with 144591.2 person years. Thus excluding missing answers resulted in an incidence rate per 1000 person years of 2.32 (95% CI 2.08, 2.58).

## Discussion

In this national representative study group, we found a beneficial effect of lean fish consumption on T2DM. Consumption of 75–100 g of lean fish per day, corresponding to approximately 2.5–3 large servings per week, was associated with a reduced risk of T2DM by close to 30%, compared to zero consumption. It is important to emphasize that lean fish here refers to boiled or fried whole fish or filets of cod, saithe, haddock or pollock. Processed fish products containing lean fish as well as flour, milk and fat, had no effect on T2DM, nor had consumption of fatty fish, total fish intake or cod liver oil supplements.

It remains unclear whether lean fish in itself has a protective effect on T2DM or that lean fish consumers have a protective life-style that we were not able to take into account in the present study. However, dietary patterns have previously been assessed in the NOWAC cohort where fish eaters were characterized by a high intake of lean and fatty fish and fish products, potatoes, carrots, boiled coffee and fat and sauce for fish [23]. Additionally, they were mainly residing in the north and west of Norway, had lower education, were older and had higher BMI than subjects belonging to the other dietary clusters. A large proportion was also former or current smokers, thus, fish eaters were not immediately characterized by a healthy lifestyle. Nevertheless, the amino acid taurine is likely to be present in higher concentrations in cod than in farmed salmon [24]. Taurine has showed protective effect on CVD [25] and is suggested to have beneficial effect on the development of metabolic syndrome and DM [26].

A protective effect from total fish consumption and intake of small and medium sized fish was observed in Japanese men, but not in women (median intake of 63 g/d in the second quartile of total fish intake) [8]. The Shanghai Women’s health study reports a beneficial effect of fish consumption among Chinese middle-aged women (median intake of fish and shellfish ∼40 g/d) [27]. Both cohorts had a considerable lower daily intake of fish than study subjects in the present study (median intake: 83 g/d) and none of them distinguished between fatty and lean fish. In a large case-cohort study across eight European countries, no effect of total fish consumption on T2DM was observed, however they found a weak beneficial effect of fatty fish intake [11]. In that study, the median intake of fish and shellfish in the highest intake group were approximately 73 g/d, thus also lower than in the present study group. Positive associations between total fish consumption and T2DM have been reported in three papers [5]–[7], two from the United States comprising four different cohorts and one from the Netherlands. The studies from the United States explored total fish intake, whereas the Dutch study also evaluated fatty and lean fish separately, but with significant result for total fish only. In the present study, we clearly demonstrate that discriminating between different types of fish is crucial when assessing the effect of fish consumption on T2DM. As types of fish consumed vary across countries, comparing total fish intake only, may lead to misinterpretations. Cooking methods and side dishes should also be taken into account as pointed out be Patel et al [11].

Fish, and especially fatty fish, is a considerable source of n-3 fatty acids for humans. Fatty fish also contain fat-soluble persistent organic pollutants (POPs) which are anthropogenic compounds that may affect human health. The fact that fatty fish is both a source of nutrients and POPs have led to debates about the benefits and risks of fatty fish consumption [28]. Some experimental studies have raised concerns about increased risk of metabolic syndrome and T2DM after consumption of fatty fish due POPs [29]. We have previously demonstrated that fish and seafood is a source of POPs for women in the NOWAC cohort, even though only marginal differences in contaminant burden between high and low consumers of total seafood was observed [30], [31]. It is therefore important to clearly point out that no unfavorable effects of any kind of fish consumption or use of cod liver oil supplements on T2DM was observed in this study. As humans have a much more variable diet than test animals, direct comparisons to test animals fed on a specific diet (e.g. salmon) is of limited validity. Additionally it is important to emphasize that lean fish has negligible concentrations of POPs in the muscles as compared to fatty fish.

The prevalence of DM (all forms) in NOWAC was 2.6%, which is considered low on a global scale. In the United States, the corresponding number for women above 20 years of age is 10.8% [32] and in Denmark the prevalence of DM among women were 4.07% in 2007 [33]. In 2004, a prevalence study of DM in Norway was published, summarizing data from several public health surveys [34]. Among women in the age group 40–69 years the average prevalence varied from 1.1% to 3.2%, reflecting the same low occurrence as found in this study. The age-adjusted overall incidence of 2.41 per 1000 person years in the current study (mean age at baseline 48 years) is however similar to what has been reported in Denmark (3.0/1000 person years for women age 50) in 2004 [33]. In this study, we treated missing information on diabetes status as a negative answer. When conducting a sensitivity analyses on the incidence rates and removing all subjects with missing answers on self-reported DM, there was no significant difference in calculated incidence rates (overlapping confidence intervals). Additionally, results from our validation study suggest that missing answer on diabetes status can be interpreted as a negative response. This is further supported by a test-retest study on disease reproducibility in NOWAC (un-published data). We therefore considered the risk of overestimation of T2DM cases in the prospective analyses as very limited.

The aim of the cross-sectional validation study was to evaluate the positive predictive value of the NOWAC questionnaire in regards to T2DM. For all forms of DM the PPV were 93%, which we consider good. For T2DM the corresponding number was 84%. Thus, there was a 16% risk of overestimating T2DM cases, where 8% corresponded to T1DM. Including a pre-defined criteria of age at diagnosis >35 years will not improve the PPV as the self-reported mean age at time for diagnosis were 44 years among those claiming having T1DM and 48 years for T2DM cases. In a prospective setting the percentage of misclassification/overestimation of T2DM cases are likely to be less than obtained in this cross-sectional study as more than one questionnaire is available and prevalent cases at baseline can be excluded. Additionally we found that T2DM comprised 89% of DM, which is in line with previous findings worldwide [35]. We conclude that in the NOWAC study which comprise middle-aged women only, a positive answer on self-reported diabetes are likely to reflect T2DM. In order to identify T1DM cases, contact with the subjects GP is necessary.

The NOWAC study is a national representative cohort, which brings the advantage of incidence and prevalence calculations. Additionally, the NOWAC FFQ contains detailed information on various types of fish consumed, which generates a trustworthy measure of exposure. There are however a few limitations with the present study; we had no information about family history of diabetes and was not able to adjust the analyses for that risk factor. Furthermore, we had no ascertainment that those subjects reporting not having diabetes actually did not have the disease. The detailed FFQ on fish consumption may increase the risk of over-reporting fish intake.

## Conclusion

Lean fish consumption of 75–100 g/d had a beneficial effect on T2DM. Unfavorable effects of fatty fish consumption or use of cod liver oil supplements on T2DM were not observed.
